# Evaluation of TNF-α genetic polymorphisms as predictors for sepsis susceptibility and progression

**DOI:** 10.1186/s12879-020-4910-6

**Published:** 2020-03-14

**Authors:** Anca Meda Georgescu, Claudia Banescu, Razvan Azamfirei, Adina Hutanu, Valeriu Moldovan, Iudita Badea, Septimiu Voidazan, Minodora Dobreanu, Ioana Raluca Chirtes, Leonard Azamfirei

**Affiliations:** 1 Infectious Diseases Clinic, George Emil Palade University of Medicine, Pharmacy, Science and Technology of Targu Mures, 38 Gh. Marinescu St, 540139 Targu Mures, Romania; 2 Genetics Laboratory, Center for Advanced Medical and Pharmaceutical Research, George Emil Palade University of Medicine, Pharmacy, Science and Technology of Targu Mures, 38 Gh. Marinescu St, 540139 Targu Mures, Romania; 3 Johns Hopkins School of Medicine, Johns Hopkins University, 733 N Broadway, Baltimore, MD 21202 USA; 4 Immunology Laboratory, Center for Advanced Medical and Pharmaceutical Research, George Emil Palade University of Medicine, Pharmacy, Science and Technology of Targu Mures, 38 Gh. Marinescu St, 540139 Targu Mures, Romania; 5 Department of Anesthesiology and Intensive Care, George Emil Palade University of Medicine, Pharmacy, Science and Technology of Targu Mures, 38 Gh. Marinescu St, 540139 Targu Mures, Romania; 6 Department of Epidemiology, George Emil Palade University of Medicine, Pharmacy, Science and Technology of Targu Mures, 38 Gh. Marinescu St, 540139 Targu Mures, Romania

**Keywords:** TNF-α, Single nucleotide polymorphism, Sepsis

## Abstract

**Background:**

The goal of the study was to evaluate a potential role for tumor necrosis factor alpha (TNF-α) genetic variability as biomarker in sepsis. In particular, we aimed to determine if single nucleotide polymorphisms (SNPs) of *TNF-α* gene are associated with sepsis in terms of risk, severity and outcome.

**Methods:**

We performed a prospective study on 163 adult critically ill septic patients (septic shock 65, sepsis 98, further divided in 40 survivors and 123 deceased) and 232 healthy controls. Genotyping of *TNF-α* SNPs (-308G/A, -238G/A, -376G/A and +489G/A) was performed for all patients and controls and plasma cytokine levels were measured during the first 24 h after sepsis onset.

**Results:**

*TNF-α* +489G/A A-allele carriage was associated with significantly lower risk of developing sepsis and sepsis shock (AA+AG vs GG: OR = 0.53; *p* = 0.004; 95% CI = 0.34–0.82 and OR = 0.39; *p* = 0.003; 95% CI = 0.21–0.74, respectively) but not with sepsis-related outcomes. There was no significant association between any of the other *TNF-α* promoter SNPs, or their haplotype frequencies and sepsis or septic shock risk. Circulating TNF-α levels were higher in septic shock; they were not correlated with SNP genotype distribution; GG homozygosity for each polymorphism was correlated with higher TNF-α levels in septic shock.

**Conclusions:**

*TNF-α* +489G/A SNP A-allele carriage may confer protection against sepsis and septic shock development but apparently does not influence sepsis-related mortality. Promoter *TNF-α* SNPs did not affect transcription and were not associated with distinct sepsis, septic shock risk or outcomes.

## Background

The constant interest in sepsis research is justified by data showing an increased number of deaths despite a reduction in sepsis-associated mortality during the last four decades; the intra-hospital risk of death by sepsis is 10–20%, and increases to 40–80% in septic shock [1–4]. Sepsis associated mortality became higher due to the increased rates of diagnosis [3–5]. It has become more evident that major progress in reducing the morbidity and mortality associated with sepsis will only be possible by identifying early biomarkers which can be used to personalize the intervention in patients with an increased risk of sepsis and/or an unfavorable disease course.

The definition of sepsis suffered numerous changes from the initial recognition of the condition and its causality in the nineteenth century until present time as a consequence of increased understanding of the disease mechanisms. Infection is the prerequisite of sepsis, but an inefficient intervention of host factors represents the turning point in the evolution of an infection towards sepsis. The Third International Consensus Definition for Sepsis and Septic Shock (Sepsis-3) recognized a dysregulated host response as necessary for the onset of sepsis [6].

Following the formulation of Predisposition, Infection or insult, Response and Organ dysfunction (PIRO) as a patient stratification system and investigation of a predisposing role for the host genetic variability, a significant amount of evidence has accumulated on the effect of the systemic immune response genetic variability in sepsis and its repercussions on susceptibility and outcome [7–10]. The prospect of modulating the expression of cytokines and unbalancing the equilibrium of the pro- and anti-inflammatory responses prompted the interest in identifying relevant genetic variations with an effect on the sepsis risk and prognostic [11].

Tumor necrosis factor alpha (TNF-α) is at the epicenter of the systemic inflammatory response given its early and deterministic role in releasing other cytokines, as well as its direct functional effects in septic shock. Its plasma levels have been correlated with sepsis mortality [12–17]. TNF-α has been involved in sepsis immunodepression through increased apoptosis [18]. The significant contribution of the TNF-α to the pathology of the inflammatory response generated the idea of a possible genetic determinism of its circulating levels and hence of sepsis risk and evolution [12, 13, 18–35]. The single nucleotide polymorphism (SNP) at position -308 in the promoter region of the *TNF-α* gene is the most studied in sepsis and has been associated with higher transcription rates of the cytokine and, more often than not, with an increased risk of sepsis [13, 18, 32, 36, 37]. These findings remain inconclusive: some authors failed to demonstrate one or both of these associations [28, 37–41]; furthermore, results associating septic shock development and mortality risks with the genetic variability of the gene are conflicting [18, 38, 41–44]. Other SNPs located in the promoter of the gene (-238G/A, -376G/A) seem to have a functional importance in TNF-α response and subsequently in its ability to augment its deleterious effects and eventually affect morbidity and mortality in sepsis [18, 38, 41, 45, 46]. Polymorphism +489G/A is located in the first intron of the *TNF-α* gene. Although it has no impact on the protein sequence, some of the few studies available so far describe a modulating role in some chronic conditions associated with inflammation (chronic obstructive pulmonary disease - COPD, autoimmune diseases); it might exert an effect by differentially influencing the mRNA stability [18, 47, 48]. Its potential effect in inflammatory reactions associated with sepsis is virtually unknown - hence our interest in studying it.

We performed a controlled study to investigate the impact of *TNF-α* gene polymorphisms, namely *TNF-α* -308G/A (-308G>A), *TNF-α* +489G/A (+489G>A), *TNF-α* -238G/A (-238G>A), *TNF-α* -376G/A (-376G>A), on sepsis risk, septic shock development and outcome; correlations were sought between allele, genotype and haplotype distribution and severity of disease. We also examined the relationship between *TNF-α* genetic variability and its circulating levels.

## Methods

### Study population

This study was conducted between September 2015 and June 2017 in the Intensive Care Unit (ICU) of the Emergency Clinical County Hospital Targu Mures, Romania, which is an extended competence reference unit of the highest rank: critically ill patients with complex pathologies are referred to it from regional hospitals and other ICUs. The department is a 32-beds mixed, surgical and medical ICU, which also serves a university hospital and has a yearly turnover of 1700 patients [49].

The study was designed as a non-interventional prospective cohort study. The patients enrolled during the first 10 months of the study had been included in a previous report discussing interleukin *IL-6* genetic variability in sepsis [50]. The previously reported inclusion and exclusion criteria were applied. Briefly, Caucasian patients over 18 diagnosed with sepsis were enrolled unless they had confounding conditions such as neoplasia, HIV infection, or autoimmune disorder necessitating immunosuppressive drugs. All patients coming from medical or surgical departments who were admitted to the ICU of the above mentioned hospital were evaluated and enrolled in the present research if the sepsis onset occurred during the previous 24 h. Sepsis was defined in accordance to Levy et al. as confirmed or suspected infection and at least two systemic inflammatory response syndrome (SIRS) criteria [7, 51]. Arterial hypotension (when the systolic blood pressure was below 90 mmHg or the mean arterial pressure was below 70 mmHg that was refractory to appropriate volume repletion) associated to sepsis was defined as septic shock [52]. A Caucasian control group consisting of 232 healthy adult individuals with a similar age and sex distribution were included in the study. The same pathologies were selected as exclusion criteria for the control group.

The Ethics Committees of the participating Targu Mures County Hospital and of the University of Medicine and Pharmacy of Targu Mures, Romania approved the study protocol. Informed written consent was obtained from healthy controls, patients or patients’ next-of-kin (for unconscious patients. All samples were anonymized to ensure patient confidentiality.

### Clinical monitoring evaluation

The following variables were recorded: a) clinical data – consisting of number of hospitalization days in the ICU, day of death (if applicable), days of vasoactive therapy and mechanical ventilation, comorbidities; b) severity score upon enrollment in the study (Acute Physiology and Chronic Health Evaluation APACHE II, Simplified Acute Physiology Score SAPS II and Sequential Organ Failure Assessment SOFA); c) anthropometric and biological data.

### Blood sampling

Two venous blood samples were collected in ethylenediaminetetraacetic acid (EDTA) coated tubes from all enrolled patients during the first 24 h following sepsis diagnosis. One whole blood sample used for DNA isolation was collected and processed immediately; a second blood sample was centrifuged at 1000 relative centrifugal force (RCF) for 10 min; the obtained plasma was aliquoted and stored at − 80 °C until processing.

### SNPs genotyping

Genomic DNA was isolated from blood samples using the Quick-gDNAMiniPrep kit (ZymoResearch, Irvine, USA). SNP Genotyping was achieved through Real-Time PCR amplification using TaqMan® technology for *TNF-α* (-238G/A, rs361525 and -376G/A, rs1800750) on the Applied Biosystems *7*500 Fast Real*-*Time PCR System. Results were analyzed using the TaqMan® Genotyper Software. *TNF-α* +489G/A genotyping analysis was performed by polymerase chain reaction–restriction fragment length polymorphism (PCR-RFLP) as previously described by Kothari et al. [18]; *TNF-α* -308G/A gene polymorphism was analyzed by the ARMS-PCR (Amplification-refractory mutation system - PCR) technique [52].

### Haplotype analysis of TNF-α -238G/A, -308G/A and -376G/A gene polymorphism

Haplotype analysis of investigated *TNF-α* gene polymorphisms was performed using Haplotype analysis – software version 1.05 available at Georg-August-Universität Göttingen [53].

### TNF-α measurement

TNF-α plasma levels were determined using the xMAP technology (Luminex Corporation, Austin, USA) and the TNF-α Human Magnetic Luminex Assay (R&D Systems Ref. LXSAHM, Minneapolis, USA). The sensitivity of the assay was 1.2 pg/mL. The samples were incubated with magnetic beads coupled to anti-TNF-α monoclonal antibodies. During the second step, antigen-antibodies complexes were incubated in a biotinylated antibody solution, followed by Streptavidin-Phycoerythrin. Removal of unbound reagents was performed after each step. Antigen-antibodies sandwich complexes were interrogated through dual-laser fluorescence excitation using the Luminex 200 platform (Luminex Corporation, Austin, USA). The concentration of TNF-α was determined using the Median Fluorescence Intensity (MFI) and five parameters logistic curves.

### Statistical analysis

Statistical Package for Social Sciences (SPSS, version 20, Chicago, IL, USA) was used to carry out the statistical analysis for the present study. Distribution normality of quantitative variables was tested by using the Kolmogorov-Smirnov test and descriptive statistics (mean, median, SD, IQR) were calculated when necessary. Based on the results of the normality test and the number of groups, quantitative variables were compared across our study by using either the Kruskal–Wallis test, Mann-Whitney test or t-test. Contingency tables and the chi-square test were used to examine associations between genotype distribution and other categorical variables. The probability or susceptibility to sepsis based on the given polymorphisms was determined through OR (odds ratio) calculations. A Chi-square test was used to calculate deviations of allelic frequencies from Hardy–Weinberg equilibrium. Using simple binary logistic regression, we analyzed each polymorphism as a possible predictor for sepsis. The significance level threshold was set at α = 0.05; measures below the significance threshold were considered statistically significant. Power calculations for the Chi-square test were performed with G* Power v. 3.1.9.4 [54].: 1-beta error probability was 0.78.

## Results

### Overall demographic and clinical characteristics of patients

The patient cohort comprised a total of 163 patients, who were further divided into two subgroups, namely septic shock (SS) (*n* = 65, 39.87%) and sepsis (S) (*n* = 98, 60.12%), according to the criteria previously mentioned. Demographic, clinical and outcome data are summarized in Supplemental Table 1. Mean patient age was 64.9 ± 14.3 years, with the sex distribution favoring males (95/68, *p* = 0.026); these parameters were matched within the control group. For further statistical analysis, the study group was subdivided based on outcome in survivors (40; 24.5%) and deceased (123; 75.5%).

### TNF-α plasma levels

Plasma TNF-α levels were significantly higher in the septic shock (SS) patient subgroup (median 57.3 pg/mL; range: 10.21–207.5 pg/mL) compared to the sepsis (S) subgroup (median 42.9 pg/mL; range: 10.2–207.1 pg/mL; *p* = 0.0007).

Further analysis revealed circulating TNF-α level to be significantly increased in septic patients who died while in the ICU (53.71 pg/mL; range: 10.21–207.5 pg/mL) as compared to survivors (median 39.68 pg/ml; range: 10.21–100.6 pg/ml, *p* = 0.02).

### Genotypes and allelic distribution of the studied SNPs

For *TNF-α* -308G/A, *TNF-α* -238G/A, *TNF-α* -376G/A and *TNF-α* +489G/A SNPs, the genotypes distributions were consistent with Hardy-Weinberg equilibrium (HWE) in both study and control groups. We were unable to identify differences according to age, gender and body mass index (BMI) between patients with variant genotypes of the studied polymorphisms (Supplemental Table 2).

The genotypes and allelic distributions of the investigated *TNF-α* SNPs in the septic shock and sepsis subgroups as well as in controls are presented in Table 1 for +489G/A SNP and in Supplemental Table 3 for the other studied SNPs).

**Table 1:** Genotype and allele frequencies of *TNF-α* +489G/A gene polymorphisms in control and septic patient group and subgroups

Genotype/ allele	Study group n (%)	Septic shock n (%)	Sepsis n (%)	Control n (%)	*p*^a^; OR (95% CI)	*p*^b^; OR (95% CI)	*p*^*c*^; OR (95% CI)
AA	3 (1.84)	0 (0.0)	3 (3.1)	15 (6.5)	0.01; 0.22 (0.06–0.8)	0.26; 0.18 (0.009–3.72)	0.01; 0.08 (0.009–0.44)
AG	44 (26.99)	15 (23.1)	29 (29.6)	85 (36.6)	0.01; 0.58 (0.37–0.91)	0.36; 0.68 (0.33–1.40)	0.01; 0.46 (0.24–0.88)
GG	116 (71.16)	50 (76.9)	66 (67.3)	132 (56.9)	Reference	Reference	Reference
AA + AG	47 (28.83)	15 (23.1)	32 (32.7)	100 (43.1)	0.004; 0.53 (0.34–0.82)	0.21; 0.61 (0.30–1.26)	0.003; 0.39 (0.21–0.74)
Allele G	276 (84.7)	115 88.5)	161 (82.1)	349 (75.2)	Reference	Reference	Reference
Allele A	50 (13.33)	15 (11.5)	35 (17.9)	115 (24.8)	0.001; 0.54 (0.38–0.79)	0.12; 0.60 (0.31–1.15)	0.001; 0.39 (0.22–0.70)
HWE Test	0.61	0.29	0.93	0.68			

*OR* Odds ratio, *CI* Confidence interval, *HWE* Hardy-Weinberg equilibrium

*p*^a^: *p* values for individual genotypes in study group vs control; *p*^b^: *p* values for individual genotypes in sepsis subgroup vs septic shock subgroup; *p*^c^: *p* values for individual genotypes in septic shock subgroup vs control

### Effect of SNPs on TNF-α plasma levels

**Fig. 1:**
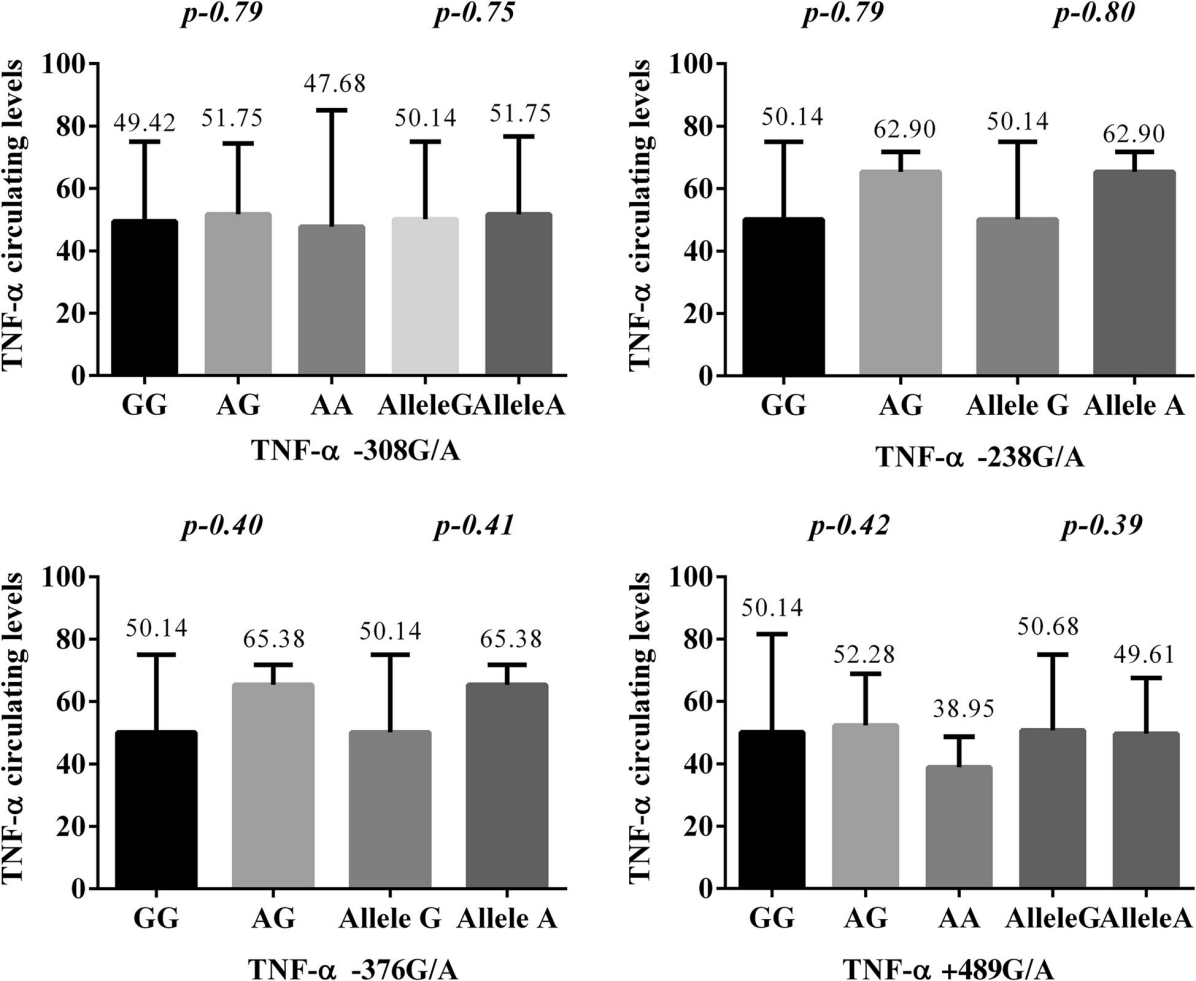
Plasma TNF-α levels in genotype polymorphisms. Values are expressed as median (interquartile range). *p* values were calculated separately for genotypes and alleles

The main observation across the entire sepsis patient study group was that plasma TNF-α levels were higher in patients with the heterozygous genotypes of *TNF-α* -238G/A and *TNF-α* -376G/A compared to the wild type (WT) homozygous genotype; the A allele was associated with increased concentration expression of the cytokines, though without reaching statistical significance (Fig. 1). No differences were observed in the plasma concentrations of TNF-α between any of the studied SNPs for the entire sepsis patient group or any of the subgroups (Fig. 1, Table 2). Logistic regression analysis of the alleles and genotypes of the four SNPs revealed no association with modified plasma levels of the TNF-α (*p* > 0.1).

**Table 2:** TNF-α circulating levels^a^ according to the genotypes of investigated *TNF-α* polymorphisms

	Genotype	Sepsis subgroup	Septic shock subgroup	*P* value
*TNF-α *-308G/A	GG	41.31 (10.21–207.1)	57.28 (10.21–207.5)	0.01
	AG	44.09 (20.14–141.5)	57.28 (12.48–186.3)	ns
	AA	47.68 (10.21–85.14)	–	–
	AA + AG	44.09 (10.21–141.5)	57.28 (12.48–186.3)	ns
Allele	G	42.94 (10.21–207.1)	57.28 (10.21–207.5)	0.0007
	A	44.09 (10.21–141.5)	57.28 (12.48–186.3)	0.07
*TNF-α *-238G/A	GG	42.58 (10.21–207.1)	57.28 (10.21–207.5)	0.008
	AG	62.2 (20.14–72.69)	63.6 (44.39–73.39)	ns
Allele	G	42.9 (10.21–207.1)	57.3 (10.21–207.5)	0.0005
	A	62.2 (20.14–72.69)	63.6 (44.4–73.4)	0.57
*TNF-α *-376G/A	GG	42.58 (10.21–207.1)	57.28 (10.21–207.5)	0.002
	AG	–	63.6 (44.39–73.39)	–
Allele	G	42.9 (10.21–207.1)	57.3 (10.21–207.5)	0.0005
	A	67.5 (10.21–54.2)	63.3 (44.4–73.4)	0.30
TNF-α +489G/A	GG	44.09 (10.21–207.1)	57.09 (10.21–207.5)	ns
	AG	33.45 (10.21–175.2)	59.04 (26.76–147.1)	ns
	AA	38.95 (25.26–48.71)	–	–
	AA + AG	36.2 (10.21–175.2)	59.04 (26.7–147.1)	ns
Allele	G	43.3 (10.21–207.1)	57.3 (10.21–207.5)	0.001
	A	36.2 (10.21–175.2)	59.04 (26.7–147.1)	0.01

*ns* Non-significance

^a^Plasma level (pg/mL), expressed as median (range), *p*: Septic shock group vs sepsis subgroup, Mann-Whitney test

We subsequently compared plasma levels of TNF-α in the two subgroups for each polymorphism genotype. Plasma levels of TNF-α were significant higher in shock compared to septic patients for the GG homozygous genotypes in the case of the *TNF-α* -308G/A, -238G/A, and -376G/A SNPs (Table 3). Moreover, G carriers of all aforementioned SNPs, but also of +489G/A had significantly higher production values of circulating TNF-α and were more likely to develop septic shock; additionally, in patients who developed septic shock the A allele of SNP + 489 was associated with significantly higher circulating levels of TNF-α (Table 2).

**Table 3:** Logistic regression analysis for genotype and allele frequency of *TNF-α* +489G/A SNP in study group and both subgroups compared to controls model

	Variable	Odds Ratio	95% CI	*P* value
Sepsis vs. Controls	GA	0.72	0.43–1.21	0.21
	AA	0.45	0.13–1.61	0.22
	AA+AG	0.64	0.38–1.05	0.07
	Allele A	0.64	0.38–0.05	0.07
Septic Shock vs. Controls	GA	0.51	0.27–0.97	0.04
	AA	0.06	0.24–3.98	0.98
	AA+AG	0.39	0.21–0.74	0.004
	Allele A	0.39	0.21–0.74	0.004
Sepsis and Septic shock cumulated vs. Controls	GA	0.64	0.41–0.98	0.04
	AA	0.27	0.07–0.95	0.04
	AA+AG	0.53	0.34–0.82	0.004

### The impact of the investigated TNF-α SNPs on sepsis risk

Our results found +489G/A to be the only SNP that correlated with sepsis risk (Table 1). We identified a significant difference in the variant genotype frequency of *TNF-α* +489G/A: the frequency of the AA genotype was 3.5 times larger in the control group than in the study group (OR = 0.22; 95% CI = 0.06–0.80, *p* = 0.01). This SNP, namely *TNF-α* +489G/A was associated with a diminished risk of sepsis under the dominant model (GA + AA vs GG, OR = 0.53; 95% CI = 0.34–0.82, *p* = 0.004) and allelic comparison (A vs G, OR = 0.54; 95% CI = 0.38–0.79, *p* = 0.001) (Table 1). The association is supported by logistic regression analysis, the findings suggesting that the presence of the A allele was significantly associated with a lower sepsis risk (*p* = 0.004). All the results are strongly suggestive for a protective role of the minor allele frequency (MAF) A of +489G/A SNP against sepsis susceptibility (Table 3).

None of the SNPs in the *TNF-α* promoter, namely -308G/A,  -238G/A and -376G/A, showed an association with the risk of sepsis, although the frequency of the homozygous AA genotype of -308G/A SNP and respectively, of the heterozygote genotypes of all the three SNPs (no homozygous AA genotypes for  -238 and  -376 SNPs were found in the group) was increased in the sepsis group compared to controls (Supplemental Table 3).

### The effect of the investigated TNF-α SNPs on septic shock development and mortality

The comparison of genotype and allele frequencies between septic shock subgroup and control group reveals significant differences for SNP +489G/A. Having at least one A allele conferred a protective role against the development of septic shock (AA+AG vs GG; *p* = 0.003; OR = 0.39; 95% CI = 0.21–0.74); the differences were also significant under all the other studied genetic models (homozygote AA vs GG, heterozygote AG vs GG and allelic comparison A vs G) (Table 1).

No significant differences were found in allele and genotype frequencies between the septic shock and septic patients. However, AA variant homozygous genotypes of the *TNF-α* +489G/A were found exclusively in septic patients and AG heterozygosity was also higher in this subgroup (Table 1).

Following a univariate logistic regression analysis, MAF A allele carriers of this SNP (*p* = 0.004; OR = 0.396; 95% CI = 0.210–0.746), as well as AA and GA variant genotypes, were significantly associated with a lower septic shock risk (Table 3). No significant differences between the study groups and subgroups for the other three SNPs were found (Supplemental Table 3).

When outcome in the entire septic study group was examined we also identified one difference at the edge of statistical significance and only under the dominant model of the *TNF-α* -308G/A SNP; mortality was reduced in A allele carriers (AA+AG vs GG; *p* = 0.05; OR = 2.22; 95% CI = 1.01–4.88) (Table 4). No allele or genotype of the four SNPs showed associations with the severity scores across the entire sepsis group or separately across subgroups.

**Table 4:** Allele and genotype frequencies of *TNF-α* -308G/A, -238G/A, -376G/A and +489G/A SNPs according to outcome in the study group

	Survivors n (%) 60 (42.9%)	Deceased n (%) 103 (57.1%)	Total n (%) 163	*P* value; OR (95% CI)
*TNF-α* -308G/A				
AA	1 (1.7%)	1 (1.0%)	2 (1.2%)	0.88; 1.90 (0.11–31.26)
AG	16 (26.7%)	20 (19.4%)	36 (22.1%)	0.32; 1.52 (0.71–3.24)
GG	43 (71.7%)	82 (79.6%)	125 (76.7%)	Reference
AA+AG	17 (28.3%)	21 (20.4%)	38 (23.3%)	0.25; 1.54 (0.73–3.23)
*TNF-α* -238G/A				
AG	3 (5.0%)	3 (4.9%)	8 (4.9%)	0.67; 1.71 (0.33–8.80)
GG	57 (95.0%)	98 (95.1%)	155 (95.1%)	Reference
*TNF-α* -376G/A				
AG	0 (0.0%)	4 (3.9%)	4 (2.5%)	0.29; 0.18 (0.009–3.45)
GG	60 (100.0%)	99 (96.1%)	159 (97.5%)	Reference
*TNF-α* +489 G/A				
AA	2 (3.3%)	1 (1.0%)	3 (1.8%)	0.56; 3.15 (0.27–35.84)
AG	13 (21.7%)	31 (30.1%)	44 (27%)	0.35; 0.66 (0.31–1.39)
GG	45 (75.0%)	71 (68.9%)	116 (71.2%)	Reference
AA+AG	15 (25.0%)	32 (31.1%)	47 (28.8%)	0.47; 0.74 (0.36–1.51)

### Clinical relevance of the haplotypes with sepsis, septic shock development, and mortality

We included in the haplotype analysis polymorphisms located in the promoter of the *TNF-α* gene, namely -308G/A, -238G/A and -376G/A and we investigated if haplotypes are associated with sepsis. The most frequent *TNF-α* -308G/A, -238G/A and -376G/A haplotypes were as follows: GGG (85.5%), AGG (11.21%), GAG (1.24%) in sepsis study group and GGG (87.28%), AGG (9.42%), GAG (1.5%) in controls (*p* > 0.05). All other haplotypes identified (GGA, GAA, AGA, AAG, and AAA) had frequencies under 1% in both study group and controls. Haplotype analysis correlated to hospital outcome in the studied group showed that the presence of the A allele in the -308G/A SNP together with the WT allele in the -238 and -376 positions has a protective effect against sepsis mortality; haplotype AGG was identified more frequently among survivors than deceased (18.12% vs 23.25%, *p* = 0.046). We also performed a logistic regression and found the A allele to be protective with regards to outcome, and increasing age and SAPS score to be associated with mortality. After adjusting for the last two mentioned variable elements, there was no significant association of this allele or other studied SNP genotypes and alleles with ICU mortality.

## Discussion

Various studies have attempted to evaluate the possibility that genetic variability of cytokines might lead to differences in immune responses with impact on sepsis susceptibility and severity and some of them indicated a role for TNF-α in generating and promoting the inflammatory response in systemic infection [42, 55]. Polymorphisms in the promoter region seem to have an increased association with the development of sepsis and septic shock in some population groups, due to differentiated gene transcription [56]. Still, published results fail to reach consensus.

A gradual increase of TNF-α plasmatic levels in sepsis was registered, with maximal values in septic shock; this was in agreement with previous studies and supports the prognostic biomarker role generally conferred to this cytokine [18, 57]. Moreover, we noticed a significantly increased concentration of TNF-α in the group of patients that subsequently deceased. This finding confirms the correlation between the levels of cytokine and disease severity and mortality [17, 18, 25, 38, 58].

Our study shows that genetic variation in the first intron of the *TNF-α* gene to have an important impact on the susceptibility to sepsis and its severity. We observed a significantly higher frequency of individuals who are homozygous for the A allele and also of +489 SNP heterozygotes in the control group than in the group of septic patients; this association was also noticed for comparisons with each subgroup as well (sepsis, septic shock). Only few studies examined the risk for sepsis associated with this newly described SNP [59]. Our results differ from previous observations on the association of +489G/A SNP with sepsis and septic shock or other pathologies: Kothari et al. found an association between +489G/A SNP and development of severe sepsis and septic shock in critically ill patients from ICUs [18, 47]. A protective role was attributed to the A allele in relation to the susceptibility, severity and evolution of rheumatoid arthritis, albeit the results were not confirmed [60, 61]. We report a frequency of allele A of 0.25, similar to that reported by Ensemble genome browser [62] but higher than the ones reported in other European countries, which vary according to the reports from 0.08–0.15 in England, to 0.12 in Holland, 0.13 in Spain and 0.12–0.139 in Italy [59–61, 63]. By extrapolation, this might seem to indicate a decreased sepsis susceptibility in the Romanian population, but our sampling strategy did not seek to be representative. The observation that the +489G/A SNP confers a protective role against sepsis and septic shock was not confirmed when we examined the outcomes: the survival rates were not influenced by this allele distribution.

Several SNPs in the promoter of *TNF-α* gene have been previously linked to sepsis risk, including -308G/A, -376G/A and -238G/A [13, 18, 25, 30, 36, 44, 45, 64]. Kothari et al. suggested a relationship of all these SNPs with sepsis through regulation of *TNF-α* gene transcription and suggested genotyping ICU patients for personalized, genetically tailored therapies [18].

This study did not find a significant association between any of the promoter SNPs and an increase in sepsis susceptibility in ICU critical patients. This is in line with other studies that did not identify an association between the G/A genotypes of the *TNF-α* -308 and/or -238 SNPs and sepsis risk in ICU Caucasian patients [28, 40]. Similar results were reported by Gordon et al. in the case of 213 ICU patients from the UK and Australia [38] and also for -308 only in a Colombian population [39].

All the mentioned results are contradicted by findings from several other studies that point to a significant role of the less common A allele of the *TNF-α* -308 SNP in sepsis development in patients undergoing major surgery [13], in burns [30, 64], after severe trauma [45, 65] or in general ICU Caucasian or Japanese population [25, 36]. One explanation for the heterogeneity of the results can reside in the genetic differences between studied populations; however, meta-analyses that evaluated the implications of the SNPs on the risk of sepsis were themselves rather conflicting, indicating a positive association in Caucasians only [66], strongly or exclusively in the Asian population [14, 67], and in the overall population [42]. Another explanation might be that criteria used to define sepsis lack uniformity. If the 2011 criteria are used, our study fails to display a correlation between promoter SNP haplotypes and sepsis risk, as opposed to findings by Restas et al. They evidenced the protective role of minor frequency *TNF-α* SNP (including -308G/A and -238G/A) allele for some haplotypes in sepsis development, after failing to reach a similar result on the same patient group by using former definitions based on SIRS criteria [7, 68]. Unfortunately, we were unable to reclassify our patients according to Sepsis-3 definition due to the partial lack of some important data. However, on the relatively small number of patients that we managed to reanalyze there still was no correlation (data not shown).

In this study, we could not identify any associations between variant genotypes of the studied SNPs and TNF-α plasma levels in septic and septic shock patients; none of the alleles correlated with specific plasma cytokine levels.

By contrast, previous studies found significantly higher TNF-α serum concentrations in Chinese Han patients with the AA+AG genotypes, who developed severe sepsis compared to the GG homozygotes [32], and in Asian patients carrying the *TNF-α* -308A allele and in AA homozygotes that developed surgical sepsis, compared to patients carrying other genotypes [13, 37]. Additionally, in vitro studies identified increased TNF-α concentrations in healthy carriers of at least one A allele after exposure to lipopolysaccharides or after meningococcal infection [32, 41, 69]. However, other authors were unable to confirm an association between *TNF-α* -308G/A genotypes or allele and circulating levels of the cytokine in either Caucasian or Colombian adult septic patients [36, 38, 39], as well as in vitro [28, 41, 70].

Few studies exploring the association of the other studied SNPs of *TNF-α* with cytokine levels have been previously published and their results are partly contradictory. With respect to *TNF-α* -238G/A and -376G/A SNP, Kothari suggested an increased production of the cytokine for AA homozygous septic patients [18]. Although in our group the heterozygote genotypes of the two SNPs were associated with elevated TNF-α levels, the differences were not significant; a possible explanation being the relatively small number of A allele carriers. Our results were similar to those obtained by Gordon et al. in a prospective multicenter study on septic Caucasian patients that could not demonstrate enhanced TNF-α levels associated to -238G/A and -308G/A SNPs [38].

When comparing the median TNF-α plasma levels between the two study subgroups, we noticed a statistically significant difference in the case of all four polymorphisms only within the population of GG genotypes and G allele carriers. This finding would appear to implicate a possible proinflammatory role of *TNF-α* SNP G alleles, manifested as hyperstimulation of the immune response in sepsis onset [71]. However, we cannot exclude that this difference may lack biological significance, being just an epiphenomenon caused by the low prevalence in our group of genotypes including the A alleles. Previously, and in line with our results, Retsas et al. demonstrated that patients carrying the WT allele of several SNPs, including the -308G/A and -238G/A haplotypes, have higher circulating levels of TNF-α, and suggested that minor SNP alleles could thwart the increase in cytokine production observed in individuals carrying the predominant allele [68].

Our finding regarding the lack of association of -308G/A genotype variants with septic shock or disease severity is consistent with other results reported on ICU septic patients [37, 40]. Contradictory results have been obtained in other studies on septic ICU patients [18, 36] in severely injured trauma patients and in burn victims [30, 45]; this SNP was also associated with the duration of mechanical ventilation as a marker of severity in critical patients [72]. Recent meta-analyses also led to conflicting results: Wang et al. concluded that -308G/A SNP is significantly related to septic shock risk under the dominant model, but Zhang et al. failed to demonstrate this association in a stratified analysis of severe sepsis and septic shock [14, 66].

We also evaluated the genotype distribution in the septic shock subgroup as related to that of sepsis. Our data do not support a role in sepsis severity. Few reports evaluating the differences in genotype distribution of -308G/A SNP between septic shock and sepsis had discriminating results by finding that A allele carriers of this SNP have an increased risk of developing septic shock compared to major G allele homozygotes [21, 32, 45, 64]. Cardoso et al. did not find an association and argued for the lack of a role for the -308G/A SNP in predisposing to septic shock [73]. Additionally, other authors could not detect any changes in its allelic or genotypic distribution in patients that developed septic shock versus sepsis [43, 74]. We found a slightly lower rate of mortality in patients carrying at least one A allele of -308G/A SNP, at the border of statistical significance, *p* = 0.05) within the entire group of septic patients. Several authors found contrasting results, which indicate this allele to be strongly associated either with septic shock risk and mortality [36, 37, 45] or with sepsis risk and mortality [25, 33]; some of them have even suggested to include this SNP in severity scores [33].

Several studies [43, 74] did not associate -308G/A SNP with the life prognosis, neither did the vast majority of studies which failed to identify any association of this SNP and sepsis risk [21, 28, 32, 38, 40, 41, 61, 73]. The association was also rescinded by studies which identified the minor A allele as a risk factor for meningococcal and postoperative sepsis [13, 69]. Recent meta-analyses do not confirm any impact of the SNP genetic variations on mortality [14, 66, 67].

Our result is similar to the one obtained by Surbatovic et al., who identified the AA genotype to be correlated with an increased survival rate in Caucasian critical patients, with or without sepsis [12]; also, Montoya-Ruiz et al. identified the GG dominant genotype as a risk factor for mortality on a group of critically ill Columbian patients [39]. However, our results which suggest a protective role of MAF A allele of -308 SNP and of the h2 haplotype (AGG) are not supported by the multivariate logistic regression analysis. This is in line with the Retsas et al. study which established that haplotypes containing this allele did not influence patient survival within a group of septic patients reclassified according to Sepsis-3 definitions; similar conclusions were previously published by Gordon et al. [38, 68, 75].

The particularly high mortality rates were however not unexpected within our study group. The severity scores in this reference unit are significantly above the levels in other ICUs. Consequently, this also leads to much higher mortality rates, which is also reflected in previous sepsis studies [50, 76, 77].

Our results on *TNF-α* -238G/A and -376G/A SNPs are in concordance with those previously reported by Kotsaki et al. that A allele was not associated with sepsis, septic shock risk or outcome [41]. For -238 G/A SNP only, Gordon et al. reached similar conclusions after a study on ICU septic patients and Solé-Violán in community-acquired pneumonia patients [38, 43]. Zhang M et al. identified the role of -238G/A SNP in sepsis risk in Caucasian population, while another recent meta-analysis concludes on a link between the SNP and sepsis and septic shock risk in Asian population, without providing evidence for its risk in sepsis in Caucasians [14, 42].

Our results differ from those obtained by Kothari et al., who observed a statistically significant difference in the distribution of genotypes for the four studied SNPs in sepsis and septic shock patients admitted to Indian ICUs, compared to healthy adults [18]. These authors also noted significant differences in TNF-α plasma levels - increased in sepsis and septic shock patients compared to SIRS and healthy controls, deducing an increased production of the cytokine in patients with -238G/A SNP. In our group of Romanian patients, we did not find an association of those genotypes with plasma levels and sepsis risk. Similar to the observation on septic shock, we noticed a significant association of sepsis risk with this -238G/A SNP, only when it is accompanied by increased plasma concentration of the cytokine as described by others as well [13, 32, 36, 69]. We found the same lack of association in studies that infirmed the association of the SNP with cytokine levels [28, 37–40]. In this context, it is possible that the intense proinflammatory effect of the A allele or of some of the SNPs is manifested in some population groups, possibly through linkage disequilibrium due to the localization of the *TNF-α* locus in the proximity of other inflammatory response genes from the major histocompatibility complex [78]. One limitation of the study is the rather small number of patients we had access to. We believe that some of our conclusions regarding variants in *TNF-α* genes still need to be validated on larger but similar groups.

## Conclusion

An important finding of the present report is the protective role of the *TNF-α* +489G/A polymorphism against sepsis and septic shock development; however, it did not influence mortality in septic adult Caucasian patients. We reconfirm that circulating TNF-α levels are significantly increased in deceased versus survivors, as well as in septic shock patients compared to sepsis, this difference being determined by the WT G allele of the studied SNPs. This study did not find a functional consequence of some polymorphisms in the promoter of the *TNF-α* gene (-238G/A, -308G/A, -376G/A) with regard to cytokine production, sepsis evolution and mortality risk.

## Supplementary information

**Additional file 1: Supplemental Table S1.** Overall demographic, detailed clinical and outcome characteristics of septic patients. **Supplemental Table S2.** Age, sex and BMI distributions in the study group according to *TNF-α* SNP genotypes. **Supplemental Table S3.** Genotype and allele frequencies of *TNF-α* -308G/C, -238G/A, -376G/A gene polymorphisms in control and septic patient group and subgroups.

## Data Availability

The datasets used and/or analyzed during the current study are available from the corresponding author on reasonable request.

## Abbreviations

APACHE II: Acute Physiology and Chronic Health Evaluation
ARMS: Amplification-refractory mutation system
BMI: Body mass index
COPD: Chronic obstructive pulmonary disease
EDTA: Ethylenediaminetetraacetic acid
HWE: Hardy-Weinberg equilibrium
ICU: Intensive care unit
IQR: Interquartile range
MAF: Minor allele frequency
MFI: Median fluorescence intensity
PIRO: Predisposition, Infection or insult, Response and Organ dysfunction
SAPS II: Simplified Acute Physiology Score
SIRS: Systemic inflammatory response syndrome
SNP: Single nucleotide polymorphism
SOFA: Sequential Organ Failure Assessment
TNF-α: Tumor necrosis factor alpha
WT: Wild type
