# Supplementary material for: Evaluation of TNF-α genetic polymorphisms as predictors for sepsis susceptibility and progression
Source: BMC Infect Dis. 2020 Mar 14;20:221. doi: 10.1186/s12879-020-4910-6 (PMC7071754; doi:10.1186/s12879-020-4910-6)
Supplement: Supplementary file 1 — Additional file 1: Supplemental Table S1. Overall demographic, detailed clinical and outcome characteristics of septic patients. Supplemental Table S2. Age, sex and BMI distributions in the study group according to TNF-α SNP genotypes. Supplemental Table S3. Genotype and allele frequencies of TNF-α -308G/C, -238G/A, -376G/A gene polymorphisms in control and septic patient group and subgroups. [file 12879_2020_4910_MOESM1_ESM.docx]

Supplemental Table 1. Overall demographic, detailed clinical and outcome characteristics of septic patients

| Variables | Septic shock patients | Sepsis patients | *p* value |
| --- | --- | --- | --- |
| BMI (Kg/m^2^), median (IQR) | 27.4 ± 0.81 | 27.3 ± 0.46 | .89 |
| ICU stay (days), median (IQR) | 5 (1-50) | 10 (1-72) | .0002*** |
| Mechanical ventilation (days), median (IQR) | 3 (0-29) | 6 (0-41) | .008*** |
| Vasoactive use (days), median (IQR) | 2 (0-27) | 1 (0-25) | .0006*** |
| Hospital mortality, n (%) | 55 (84.6) | 68 (69.4) | .04** |
| APACHE II | 28.1 ± 7.4 | 20.6 ± 7.3 | .001* |
| SAPS II | 59.7 ± 16.7 | 49.71 ± 14.7 | .001* |
| SOFA | 10.5 ± 3.5 | 7.5 ± 3.7 | .001* |
| Primary site of infection, n (%)  *Pulmonary*  *Urinary*  *Cutaneous*  *Digestive* | 35 (53.8)  7 (10.8)  13 (20)  19 (29.2) | 96 (77.6)  3 (3.1)  9 (9.2)  11 (11.2) | .002**  .045**  .048**  .004** |
| Type of admission, n (%)  *Medical*  *Surgical (scheduled)*  *Surgical (unscheduled)* | 39 (60)  2 (3.1)  24 (36.9) | 58 (59.2)  8 (8.2)  32 (32.7) | .4** |
| Comorbidities  *Arterial Hypertension*  *Chronic Ischemic Cardiomyopathy*  *Congestive Heart Failure*  *Liver Cirrhosis*  *Chronic Renal Failure*  *Obesity*  *Diabetes Mellitus* | 35 (53.8)  24 (36.9)  5 (7.7)  1 (1.5)  6 (9.2)  4 (6.2)  20 (30.8) | 40 (40.8)  30 (30.6)  6 (6.1)  1 (1.0)  2 (2.0)  4 (4.1)  15 (15.3) | .11**  .40**  .69*  .77*  .037*  .55*  .019* |

BMI: Body Mass Index, SD: Standard deviation, IQR: Interquartile range; *: Student’s test, **: Chi-square test, ***: Mann-Whitney test.

Supplemental Table 2. Age, sex and BMI distributions in the study group according to *TNF-α* SNP genotypes

| SNPs | Genotype | Number | Age | | Gender  Male/Female | BMI | |
| --- | --- | --- | --- | --- | --- | --- | --- |
|  |  |  | Mean (median/min-max) | SD |  | Mean (median/min-max) | SD (IQR) |
| *TNF-α*  -308G/A | AA | 2 | 73.00  (73/72-74) | 1.41 | 2/0 | 25.15  (25.15/24.2-26.1) | 1.34 |
|  | AG | 36 | 65.78 | 16.57 | 17/19 | 27.93 | 5.40 |
|  | GG | 125 | 64.50 | 13.70 | 76/49 | 27.19 | 5.55 |
| *p* value |  |  | *> .05* | | *.17* | *> .05* | |
| *TNF-α*  -238G/A | GG | 155 | 65.06 | 14.29 | 89/66 | 27.41 | 5.55 |
|  | AG | 8 | 61.50  (60.0/39.0-87.0) | 14.67 | 6/2 | 25.71  25.2 (20.7-31.0) | 3.54 |
| *p* value |  |  | *> .05* | | *.47* | *> .05* | |
| *TNF-α*  -376G/A | GG | 159 | 64.75 | 14.32 | 93/66 | 27.40 | 5.52 |
|  | AG | 4 | 70.50  (68.5/58.0-87.0) | 13.17 | 2/2 | 24.57  (24.1/22.5-27.7) | 2.21 |
| *p* value |  |  | *> .05* | | *.73* | *> .05* | |
| *TNF-α* +489G/A | AA | 3 | 56.00  (61.0/39.0-68.0) | 15.13 | 0/3 | 28.73  (29.4/25.7-31.1) | 2.76 |
|  | AG | 44 | 64.95 | 15.07 | 24/20 | 27.52 | 7.50 |
|  | GG | 116 | 65.09 | 14.02 | 71/45 | 27.22 | 4.58 |
| *p* value |  |  | *> .05* | | *.08* | *> .05* | |

SD: Standard deviation, BMI: Body Mass Index.

Supplemental Table 3. Genotype and allele frequencies of *TNF-α* -308G/C, -238G/A, -376G/A gene polymorphisms in control and septic patient group and subgroups.

| Genotype/  allele | Study group  n (%) | Septic shock  n (%) | Sepsis  n (%) | Control  n (%) | *p^a^/*OR  (95% CI) | *p^b^/*OR  (95% CI) | *p^c^/*OR  (95% CI) |
| --- | --- | --- | --- | --- | --- | --- | --- |
| *TNF-α* -308G/A | | | | | | | |
| AA | 2 (1.22) | 0 (0.0) | 2 (2.0) | 1 (0.4) | .56/2.94  (0.26 - 32.8) | .51/0.28  (0.013 - 5.95) | .98/1.17  (0.04 - 29.2) |
| AG | 36 (22.08) | 13 (20.0) | 23 (23.5) | 47 (20.3) | .63/1.12  (0.69 - 1.84) | .70/0.79  (0.36 - 1.71) | .95/0.97  (0.49 - 1.94) |
| GG | 125 (76.68) | 52 (80.0) | 73 (74.5) | 184 (79.3) | Reference | Reference | Reference |
| AA + AG | 38 (23.31) | 13 (20) | 25 (25.5) | 48 (20.7) | .53/1.16  (0.72 - 1.88) | .45/0.73  (0.34 - 1.56) | .66/0.86  (0.43 - 1.69) |
| Allele G | 286 (78.14) | 117 (90) | 169 (86.2) | 415 (89.5) | Reference | Reference | Reference |
| Allele A | 40 (12.26) | 13 (10) | 27 (13.8) | 49 (10.5) | .45/1.18  (0.75 - 1.84) | .31/0.69  (0.34 - 1.40) | .85/0.94  (0.49 - 1.79) |
| HWE Test | 0.74 | 0.37 | 0.90 | 0.27 |  |  |  |
| *TNF-α* -238G/A | | | | | | | |
| AG | 8 (4.90) | 3 (4.6) | 5 (5.1) | 8 (3.4) | .60/1.44  (0.53 - 3.93) | .88/0.90  (0.20 - 3.90) | .71/1.35  (0.34 - 5.26) |
| GG | 155 (95.1) | 62 (95.4) | 93 (94.9) | 224 (96.6) | Reference | Reference | Reference |
| Allele G | 318 (86.88) | 127(97.7) | 191 (97.4) | 456 (98.3) | Reference | Reference | Reference |
| Allele A | 8 (13.12) | 3 (2.3) | 5 (2.6) | 8 (1.7) | .60/1.43  (0.53 - 3.86) | .98/0.90  (0.21 - 3.84) | .71/1.34  (0.35 - 5.15) |
| HWE Test | 0.74 | 0.84 | 0.79 | 0.79 |  |  |  |
| *TNF-α* -376G/A | | | | | | | |
| AG | 4 (2.46) | 3 (4.6) | 1 (1.0) | 4 (1.7) | .72/1.43  (0.35 - 5.82) | .14/4.69  (0.47 - 46.14) | .17/2.75  (0.6 - 12.65) |
| GG | 159 (97.54) | 62 (95.4) | 97 (99) | 228 (98.3) | Reference | Reference | Reference |
| Allele G | 322 (87.97) | 127(97.7) | 195 (99.5) | 460 (99.4) | Reference | Reference | Reference |
| Allele A | 4 (2.03) | 3 (2.3) | 1 (0.5) | 4 (0.6) | .72/1.43  (0.35 - 5.75) | .30/4.6  (0.47 - 44.8) | .18/0.08  (0.004 - 1.44) |
| HWE Test | 0.87 | 0.85 | 0.96 | 0.89 |  |  |  |

*p^a^*: *p* values for individual genotypes in study group vs control; *p*^b^: p values for individual genotypes in sepsis subgroup vs septic shock subgroup; *p^c^*: *p* values for individual genotypes in septic shock subgroup vs control, OR: Odds ratio; CI: Confidence interval, HWE: Hardy-Weinberg equilibrium.
